# Effect of a Hop Extract Standardized in 8-Prenylnaringenin on Bone Health and Gut Microbiome in Postmenopausal Women with Osteopenia: A One-Year Randomized, Double-Blind, Placebo-Controlled Trial

**DOI:** 10.3390/nu15122688

**Published:** 2023-06-09

**Authors:** Manon Lecomte, Diego Tomassi, René Rizzoli, Mathieu Tenon, Thierry Berton, Sinead Harney, Pascale Fança-Berthon

**Affiliations:** 1 Givaudan France Naturals, 84911 Avignon, France; mathieu.tenon@givaudan.com (M.T.); thierry.berton@givaudan.com (T.B.); pascale.fanca-berthon@givaudan.com (P.F.-B.); 2 Biofortis, 44800 Saint-Herblain, France; diego.tomassi@biofortis.fr; 3 Service of Bone Disease, Geneva University Hospitals and Faculty of Medicine, 1211 Geneva, Switzerland; rene.rizzoli@unige.ch; 4 Rheumatology Department, Cork University Hospital, T12 DFK4 Cork, Ireland; sinead.harney@hse.ie

**Keywords:** hop, 8-PN, phytoestrogen, bone, osteoporosis, osteopenia, microbiome, menopause

## Abstract

Estrogen deficiency increases the risk of osteoporosis and fracture. The aim of this study was to investigate whether a hop extract standardized in 8-prenylnaringenin (8-PN), a potent phytoestrogen, could improve bone status of osteopenic women and to explore the gut microbiome roles in this effect. In this double-blind, placebo-controlled, randomized trial, 100 postmenopausal, osteopenic women were supplemented with calcium and vitamin D3 (CaD) tablets and either a hop extract (HE) standardized in 8-PN (*n* = 50) or a placebo (*n* = 50) for 48 weeks. Bone mineral density (BMD) and bone metabolism were assessed by DXA measurements and plasma bone biomarkers, respectively. Participant’s quality of life (SF-36), gut microbiome composition, and short-chain fatty acid (SCFA) levels were also investigated. In addition to the CaD supplements, 48 weeks of HE supplementation increased total body BMD (1.8 ± 0.4% vs. baseline, *p* < 0.0001; 1.0 ± 0.6% vs. placebo, *p* = 0.08), with a higher proportion of women experiencing an increase ≥1% compared to placebo (odds ratio: 2.41 ± 1.07, *p* < 0.05). An increase in the SF-36 physical functioning score was observed with HE versus placebo (*p* = 0.05). Gut microbiome α-diversity and SCFA levels did not differ between groups. However, a higher abundance of genera *Turicibacter* and *Shigella* was observed in the HE group; both genera have been previously identified as associated with total body BMD. These results suggest that an 8-PN standardized hop extract could beneficially impact bone health of postmenopausal women with osteopenia.

## 1. Introduction

Osteoporosis is a skeletal disorder characterized by reduced bone mass and deterioration in bone architecture leading to increased bone fragility and fracture risk [1]. Postmenopausal women are particularly at risk with approximately 30 to 40% affected by osteoporosis in the USA and Europe [2,3]. The decline in endogenous estrogen during and after menopause accelerates the bone remodeling process with an imbalance between bone formation and bone resorption [4]. In this context, phytoestrogens are an interesting non-pharmaceutical intervention to prevent bone loss. Phytoestrogens are polyphenolic plant-derived compounds with a structural similarity to endogenous human estrogen, hence their estrogenic activity. The main dietary sources of phytoestrogens are soy and red clover (isoflavones), flaxseed (lignans) and hops (prenylflavonoids) [5]. Phytoestrogens are already widely used to alleviate menopausal symptoms such as hot flashes and night sweats [6]. Moreover, isoflavones from soy and red clover have received considerable attention in the management of postmenopausal bone loss with an overall moderately beneficial effect against bone loss when consumed for at least twelve months [7,8]. There is limited literature regarding the effect of hops (*Humulus lupulus*) on bone metabolism although hops contain one of the most potent phytoestrogens known to date: 8-prenylnaringenin (8-PN).

As a novel phytoestrogen, 8-PN is unique in that its receptor specificity and potency is higher than any other phytoestrogens investigated thus far [9,10]. In vitro, 8-PN was shown to enhance differentiation and maturation of osteoblast and inhibit differentiation of osteoclast with intensities of response stronger than that observed with soy isoflavones [11]. Several in vivo studies demonstrated that an oral supplementation with a standardized hop extract was able to prevent estrogen-deficiency-induced bone loss in osteoporotic rodent models [12,13,14,15]. Moreover, in ovariectomized rats, supplementation with 68.4 mg/kg of body weight (bw) per day of 8-PN during twelve weeks improved bone biomechanical properties to the same degree as 0.7 mg/kg bw per day of estradiol, while the two other phytoestrogens tested, genistein (60 mg/kg bw per day) and resveratrol (50 mg/kg bw per day), had no significant impact [15]. From a clinical point of view, a bioavailability study performed in menopausal women indicates that prenylflavonoids (8-PN, 6-prenylnaringenin, isoxanthohumol, and xanthohumol) from a standardized hop extract are absorbed slowly, undergo enterohepatic circulation, and have long half-lives exceeding 20 h [11]. Moreover, 8-prenylnaringenin seems to be significantly more bioavailable in healthy humans than its isomer 6-prenylnaringenin [12]. In terms of health effect, three clinical studies have demonstrated the efficiency of standardized hop extract in decreasing menopausal symptoms at a dose of 100 µg of 8-PN per day during a minimum duration of 6 weeks [16,17,18]. However, to date, the potential impact of hop extract and 8-PN in the prevention of osteoporosis has not been assessed in humans.

While the presence of 8-PN in hops is low, other more abundant prenylated phenols such as xanthohumol (X) and isoxanthohumol (IX) can be metabolically converted to 8-PN. The conversion of IX into 8-PN can be accomplished enzymatically by hepatic CYP1A2 or by the gut microbiome [19,20]. However, large inter-individual variability was found for IX conversion capacity by the human gut microbiome, with only about one-third of individuals exhibiting the capability to efficiently execute this transformation [20]. *Eubacterium limosum* has been identified as one intestinal bacterium capable of facilitating the conversion (O-demethylation) of IX into 8-PN [21,22]. Finally, the gut microbiome impact on host health has been increasingly studied over past decades. There is notably a growing body of evidence indicating that the gut microbiome plays a key role in bone metabolism and osteoporosis pathogenesis even though mechanisms of action have not been clearly elucidated yet [23,24,25]. Given the role of the gut microbiome in bone health maintenance and its importance in 8-PN generation potency, it is of great interest to investigate the gut microbiome as a potential key factor in the mechanism of action of hop extract.

The present clinical trial aimed to determine whether one-year consumption of a hop extract standardized in 8-PN can moderate bone mineral density decreases in postmenopausal women with osteopenia and to explore potential mechanism of action via gut microbiome modulation.

## 2. Materials and Methods

### 2.1. Study Design and Participants

This study was a 48-week, parallel-design, placebo-controlled, double-blind, randomized clinical trial. The clinical aspects of the study were carried out from August 2019 to December 2020 in Cork (Ireland), including screening, recruitment, and follow-up. Participants were recruited through advertisements (local newspaper and social media platforms) or referred by local general practitioners in the area. A total of 221 participants were screened to identify 100 eligible participants, all of whom were postmenopausal women (>1-year post-menopause), between 50 and 85 years of age, with a body mass index (BMI) between 18–32 kg/m^2^, and presenting with osteopenia defined as a dual energy X-ray absorptiometry (DXA) T-score between −1 and −2.5 (based on the lowest T-score at any site). Exclusion criteria included osteoporosis (i.e., T-score ≤ −2.5), currently taking or had taken within the previous three months any drug for osteoporosis (bisphosphonates, parathyroid hormone, strontium ranelate, or denosumab) or any treatment with estrogen or hormone therapy or estrogen agonist/antagonist products (raloxifene or tamoxifene), had taken antibiotics or laxatives during the preceding 2 months, or had experienced gastroenteritis or foodborne illness within 4 weeks prior to the study. Participants had to be healthy, i.e., without uncontrolled hypertension, hypothyroidism, or hyperthyroidism (or must be on stable medication for at least 3 months), without history of cancer within the last five years (except basal cell carcinoma, non-squamous skin carcinoma, or carcinoma in situ with no significant progression over the past 2 years), and without significant cardiovascular, pulmonary, renal, liver, infectious disease, immune disorder, or metabolic/endocrine disorders or other disease that would preclude supplement ingestion and/or assessment of safety and the study objectives. Additionally, participants that were currently taking or had taken any vitamin K or isoflavones supplementation within the previous 4 weeks, were hypersensitive to any of the components of the investigational product (IP), were smokers, were exhibiting excess alcohol consumption, or had been trying to lose weight for the last 3 months were also excluded.

Participants who gave written informed consent and were deemed eligible at their screening visit were assigned a randomization number in chronological order. The randomization list was generated by an independent biostatistician (Atlanstat, France). A permuted-block, fixed randomization schedule was used with 2 block sizes (the first 14 blocks were size 6 and the next 14 blocks size 4), based on a computer-generated random numbers program (SAS^®^ Software version 9.4, Cary, NC, USA). All research staff involved in the collection and the analysis of the data remained blinded to the treatment randomization until all aspects of the study were complete, including the statistical analysis. A total of 50 participants were allocated to the hop extract (HE; 1 capsule per day) group, and 50 participants were allocated to the placebo group (1 capsule per day). Both groups also received calcium and vitamin D (CaD) supplements (2 capsules per day, each capsule consisting of 500 mg of calcium and 400 IU of vitamin D3; manufactured by Pharmavite, Nature Made). Participants were instructed to follow their usual dietary habits and maintain normal physical activity throughout the study, which was monitored via food and exercise questionnaires (see Section 2.6 below for details).

Randomized participants were scheduled to attend 5 visits at the research center at baseline (0), 12, 24, 36, and 48 weeks. Anthropometric parameters (height, weight, BMI, waist circumference, and hip circumference), vitals (blood pressure, heart rate, and temperature), quality of life assessed by the 36-item short form (SF-36), and physical activity assessed by the Physical Activity Scale for the Elderly (PASE) were measured at each visit. DXA measurements (BMD at femoral neck, L2–L4, total hip and total body, T-score at femoral neck and L2–L4, FRAX scores and body composition), fecal samples, and blood samples were collected at baseline, at 24 weeks, and at 48 weeks, while urine samples and dietary intake assessed by the food frequency questionnaire (FFQ) were collected at baseline and at 48 weeks only. Due to the COVID-19 pandemic and Irish government restrictions, it was not possible to conduct interim visits (weeks 12, 24, and 36) at the research center. Notably, DXA, anthropometric, and laboratory assessments were either collected on site later or were unable to be collected at these interim visits. 

The research was conducted under guidelines stated in the current revision of the Declaration of Helsinki, was approved by the Clinical Research Ethics Committee of the Cork Teaching Hospitals, and was registered at ClinicalTrials.gov (NCT04004013).

### 2.2. Study Product

Hop extract (HE) standardized in 8-PN (Lifenol^®^, Givaudan France Naturals, Avignon, France) or placebo was administrated orally in capsules. Each HE capsule comprised Lifenol^®^ containing 100 µg of 8-PN, 110 µg of 6-PN, 1.25 mg of X, and 2.94 mg of IX (measured by LC-UV) mixed with maltodextrin (Roquette Frères, Lestrem, France), and filled in red opaque gelatin capsules size 0. Placebo capsules consisted of only maltodextrin within a similar type of capsules. 

### 2.3. Bone Measurements by Dual-Energy X ray Absorptiometry

Body composition was assessed with DXA. DXA examination, performed by the same health care professionals each visit, was conducted using the Lunar iDXA ME +210575 (GE Healthcare, Chicago, IL, USA). DXA was used to determine bone mineral density (BMD) and T score at each body site; DXA was also used to determine body composition (lean mass, fat mass, visceral fat, and fat percentage). Fracture risk assessment was determined using the FRAX tool (www.shef.ac.uk/FRAX, accessed on 7 December 2020) [26].

### 2.4. Biomarkers of Bone Turnover and Biochemical Analysis

Fasting blood samples collected in EDTA/heparin tubes were centrifuged at 3000 rpm at 4 °C for 10 min within 40 min after collection. Samples were stored at −80 °C until analysis. Plasma concentrations of osteocalcin and sclerostin were measured using an automated analyzer according to the manufacturer’s instructions (Multiplex Luminex^®^ Assays, Merck-Milipore, Burlington, MA, USA). Plasma concentrations of undercarboxylated osteocalcin (uOC) (Abbexa, Cambridge, UK), collagen type 1 cross-linked *C*-telopeptide (CTx) (Abbkine, Wuhan, China), procollagen type I *N* terminal propeptide (PINP) (Abbexa), human bone alkaline phosphatase (BALP) (MyBiosource, San Diego, CA, USA), tartrate-resistant acid phosphatase isoform 5b (TRAP5b) (MyBiosource), and BALP/TRAP5b ratio were measured using ELISA according to the kit manufacturer’s instructions. The manufacturer supplied analytic variation coefficients were as follows: PINP and uOC (Abbexa): intra-assay CV < 10% and inter-assay CV < 12%; CTx, (Abbkine): intra-assay CV < 9% and inter-assay CV < 11%; BALP and TRAP5b (MyBiosource): intra-assay CV < 8% and inter-assay CV < 12%. 

Serum 25- hydroxyvitamin D (25-OH D3), plasma 17-β oestradiol, blood lipids (total cholesterol, HDL-cholesterol, LDL-cholesterol, and triglycerides), glucose homeostasis parameters (blood glucose, insulinaemia, HbA1c, and HOMA IR), and safety parameters were also measured.

### 2.5. Plasma and Urine Prenylflavonoids and Their Metabolites 

All prenylflavonoids (X, IX, 6-PN, and 8-PN) were measured both in plasma and urine in their unconjugated, glucuronide, and sulfated forms; each were expressed as a sum of the 3 different forms (i.e., total). Standard of X (purity: 99.6%), IX (purity: 99.6%), 8-PN (purity: 100%), and 6-PN (purity: 97%) were purchased from Phytolab (Vestenbergsgreuth, Germany). β-Glucuronidase enzyme from *Escherichia coli*, sulfatase enzyme from *Helix pomatia*, sodium phosphate, acetic acid, and sodium azide used for enzymatic hydrolysis were purchased from Sigma-Aldrich (Saint Louis, MO, USA). Since the concentrations measured in plasma and urine samples were very low, a calibration curve was prepared at very low concentrations ranging from 0.1 ng/mL to 5 ng/mL in methanol.

Urine and plasma samples were stored at −80 °C until analysis; they were then prepared with an automated workstation (Beckman Coulter, Biomek NX, Brea, CA, USA) in random order. For the plasma samples, 100 µL of plasma or enzymatic hydrolyzed plasma were loaded on Captiva EMR-lipid plate (Agilent Technologies, Santa Clara, CA, USA). An amount of 300 µL of MeOH/ACN (50:50) were added and mixed at 700 rpm for 3 min. Samples were then eluted by positive pressure. An amount of 100 µL of MeOH/ACN (50:50) were loaded on the cartridge once again and eluted by positive pressure. For the urine samples, 300 µL of water were loaded on Captiva ND plate, and 100 µL of urine or enzymatic hydrolyzed urine were added and mixed at 700 rpm for 1 min. Samples were then eluted by positive pressure and analyzed by LC-HRMS. Enzymatic hydrolyzed urine and plasma samples were prepared with 10 mg of enzyme diluted in 10 mL sodium phosphate buffer (100 mM, pH = 6.8) for β-Glucuronidase and in 10 mL sodium acetate buffer (100 mM, pH = 5) for sulfatase enzyme. An amount of 60 µL of this solution were mixed with 60 µL of plasma or urine and incubated during 1 h at 37 °C. Noting the difference between the concentrations measured before and after enzymatic hydrolysis provides the ability to calculate the concentrations of the glucuronide/sulfated forms. 

Liquid chromatography was performed on a UHPLC Thermo Vanquish (Thermo Scientific, Karlsruhe, Germany) in reverse phase mode with an Accucore RP-MS column (150 × 2.1 mm, 2.6 µm, Thermo Scientific) using solvent A (0.1% formic acid in water) and solvent B (0.1% formic acid in ACN). The elution gradient started at 5% B for 1 min, followed by a linear gradient rising to 90% B during 9 min. The mobile phase remained at 90% B for 5 min and then returned to initial condition after 1 min. The column was equilibrated for 4 min in initial conditions (5% B) prior to the next injection, for a total run time of 20 min. The flow rate was 0.5 mL/min, and the injection volume was 2 µL. The column was heated at 30 °C to ensure a stable column temperature and a better repeatability between runs, and the autosampler temperature was maintained at 6°C. The UHPLC system was coupled to an Orbitrap Q-Exactive Focus mass spectrometer (Thermo Scientific, Germany), and analyses were performed using an electrospray interface in negative mode, in full scan with a resolution of 35,000 FWHM in the scan range of *m*/*z* 80–1000. ESI parameters were as follows: heater temperature 300 °C, capillary temperature 350 °C, sheath gas 55 (arbitrary units), auxiliary gas 15 (arbitrary units), S-Lens 50 V, spray voltage: 3.5 kV in ESI-. Quan Browser software (Thermo Scientific) was used for quantification and the 4 targeted compounds were extracted with a mass window width of 5 ppm.

This analytical method was validated according to internal guidelines and specificity, linearity, repeatability, and limit of quantification (LOQ) are listed in Table S1. Accurate mass used for the four compounds of interest is also listed and specificity was tested by checking that matrices and diluent did not interfere with the analyte masses and retention time. Repeatability was established by analyzing 6 samples of standards solution in diluent and 6 spiked plasmas at 20 ng/mL. The relative standard deviation (RSD) of this analytical method ranged from 3.2 to 4.7% for standards solution in diluent and from 2.5 to 3.8% in spiked plasma according to the compounds (Table S1). Calibration curves also displayed satisfactory linearity with R^2^ greater than 0.99 for all compounds, and LOQ were established using a signal-to-noise ratio above 10.

### 2.6. Dietary Intake, and Physical Activity Level, and Quality of Life

Dietary habits, using the EPIC-Norfolk Food Frequency Questionnaire (FFQ; https://www.epic-norfolk.org.uk/, accessed on 7 December 2020), were evaluated at baseline and after 48 weeks. Data were entered into the Nutritics software version 5.61 (Nutritics, Dublin, Ireland), and average daily intake of total energy, fat, carbohydrate, protein, fiber, calcium, vitamin D, and isoflavones were derived.

Physical activity level was registered using the self-reported level of Physical Activity Scale for the Elderly (PASE) [27]. The PASE total score range from 0–400, where higher scores reflect a higher activity level.

Health-related quality of life was measured by measuring the means scores of the Short Form 36 (SF-36) [28]. The SF-36 is divided into eight sub-scales (physical function, role limitations-physical, bodily pain, general health, vitality, social function, role limitations-emotional, and mental health). The measurement is scored on a 0–100-point scale for each sub-scale; the higher the score, the more positive the health status.

### 2.7. Gut Microbiome Analysis

#### 2.7.1. Fecal Samples Collection and DNA Extraction

Fecal samples were collected at baseline, at 24 weeks, and at 48 weeks. Participants were provided stool collection kits and instructed to collect an at-home sample within 48 h of their next research visit. The fecal sample was collected using a collection vial and then placed immediately in the home freezer (−20 °C) before being brought to the clinic in a provided cooler bag with a cooler block. Samples received at the research center were immediately placed in a freezer at −80 °C.

Genomic DNA was extracted using the ZymoBIOMICS™ 96 MagBead DNA kit (Zymo Research Corp., Irvine, CA, USA) integrating a double lysis (mechanical and chemical) on the Precellys Evolution homogenizer (Bertin Instruments, Montigny-le-Bretonneux, France). DNA extraction was performed on the KingFisher Flex automaton (Thermo Fisher Scientific, Waltham, MA, USA) according to the manufacturer’s instructions. Once obtained, the DNA solutions were assayed by fluorimetry with the Qubit device (Thermo Fisher Scientific, Waltham, MA, USA).

#### 2.7.2. Libraries Preparation and Shotgun Metagenomic Sequencing

Fragmentation of the extracted total DNA was performed using the FS DNA Library Prep Set kit (MGI Tech, Shenzhen, China). After ligation of adapters to each sample, the libraries generated were purified on magnetic beads. Library size was verified by capillary electrophoresis on at least 10% of samples. After quantification by fluorimetry, the libraries were normalized and pooled before denaturation, single-strand circularization, and sequencing using the DNBSEQ-G400 platform (MGI Tech).

#### 2.7.3. Analysis of Overall Association and Taxonomic Profile

The MiRKAT family of tests was used to assess overall association between taxonomic compositional profiles and treatment group [29,30,31]. These are regression-based association tests based on kernels that have been proposed specifically for microbiome data and allow covariate adjustment and repeated measurements. Jaccard and Bray–Curtis beta diversity scores were used to quantify dissimilarity between compositional profiles at several taxonomic ranks. Participant sex, age, and time since menopause were added as covariates. 

Assessment of microbiota components showing differential abundance between treatment groups was evaluated using CoDA-lasso enriched with stability analysis. CoDA-lasso is a multivariate approach that fits a regularized logistic regression model with an additional constraint on the regression coefficient due to the compositional nature of relative abundance data [32]. The set of relevant taxa are those corresponding to non-zero coefficients in the solution of CoDA-lasso. Since the approach can lead to some false positives, a stability analysis was also applied to solutions from CoDA-lasso [33]. It involves refitting the model several times on independent bootstrap samples of the data and picking only those components that are selected almost always, so to delete false positives detected by chance in just a few of the re-samplings. Such stability analysis was performed using 100 bootstrap re-samplings, each comprising 80 percent of the available samples. Only components selected in at least 90% of the replicates were chosen in the final result.

The relevance of each selected taxon to discriminate between the treatment groups was investigated assessing variable importance in prediction with random forests. Each random forest comprised 500 non-pruned classification trees. Reported results comprised the selected taxa sorted according to their relative relevance in prediction, a sample estimate of the log-fold difference between the mean abundance of each group, and a heatmap of the taxa prevalence in each compared group.

#### 2.7.4. Quantification of SCFA

For the quantification of short-chain fatty acids (SCFA), fecal samples were divided in two aliquots, one for the lyophilization, and the second for a direct measure of the molecules of interest in order to obtain the dry weight-normalized absolute concentration. SCFA (acetic acid, propionic acid, butyric acid, valeric acid, caproic acid, isobutyric acid, isovaleric acid, and isocaproic acid) were measured on the second aliquot of test material via a gas chromatography–flame ionization detector (GC-FID) method as described by De Weirdt et al. [34].

### 2.8. Compliance and Adverse Events

Participants were asked to collect and return empty IP and CaD supplement containers at each visit. Compliance was calculated from the number of IP and CaD supplements returned. Compliance for both IP and CaD was calculated, in percentage, as: (100 × total number of capsules administered)/(theoretical number of capsules per day × extent of exposure in days) at each individual visit and for the entire study period. Participants were considered non-compliant for the IP if they had (1) an overall IP compliance <80% or >120%, or (2) an overall compliance within [80%; 120%], but at least one individual visit IP compliance <70% or >130%, or (3) an overall IP compliance missing and less than three available individual visit IP compliances within [80%; 120%].

Adverse events (AEs) were collected on AE forms throughout the trial. AEs were considered as treatment emergent (TEAE) if they began or worsened from the date of the first IP administration. AEs were recorded in the eCRF at each visit; recordings included a description of the AE, the relationship to the intervention (“not related” or “related or suspected”), whether the AE was serious (i.e., resulted in death, life-threatening, required hospitalization, or resulted in persistent disability) or nonserious, and the intensity of the AE (mild, moderate, severe).

### 2.9. Power Calculation and Statistical Analysis

The sample size was calculated to detect a difference in the change from baseline to 48 weeks in BMD measured by DXA on the L2-L4 lumbar spine region between HE and placebo (primary outcome) with consideration of the findings from previous studies [35,36,37]. The minimum relevant difference expected in the change in BMD (48 weeks versus baseline) between HE and placebo was 1.45% (corresponding to 0.014 g/cm^2^), and the standard deviation expected in both groups was 2.2% (corresponding to 0.021 g/cm^2^). A total of 74 evaluable patients (37 per group) were necessary to ensure an 80% power to detect a significant difference between treatment groups for two-sided test at the 5% level. Assuming 25% of non-evaluable patients, a total of 100 patients were randomized.

Data were analyzed using SAS^®^ Enterprise Guide software version 8.2 (SAS^®^ for Windows version 9.4M6). Graphs were created by GraphPad Prism version 9 (GraphPad Software, Inc., Boston, MA, USA). Statistical analyses were performed by an independent biostatistician (Atlantstat, France) on the full analysis set (FAS) according to the analysis group (HE or placebo). FAS is defined as all randomized participants with at least one dose of study treatment (HE or placebo) and with at least one not missing post-baseline value for the efficacy criteria. Due to the COVID-19 pandemic, post-baseline efficacy and safety evaluations were assigned to visits based on time windows around the planned visit dates. Only post-baseline evaluations recorded at the planned day ± 28 days were considered for the statistical analysis. Unfortunately, at week 24, due to the low number of observations for both DXA (8% and 6% for HE and placebo group, respectively) and blood collection (10% and 9% for HE and placebo group, respectively), it was not possible to conduct the statistical analysis for these parameters at this visit. No imputation rule was applied for missing values.

The primary outcome, and all other DXA parameters, were analyzed using an analysis of covariance (ANCOVA) including analysis group, baseline values, time since menopause at screening (months), and BMI at baseline (kg/m^2^). These cofactors were selected based on previous work demonstrating their relevance to bone-related outcomes in postmenopausal women [38,39]. Comparisons within and between groups were studied. The same analyses were also conducted on the relative changes from baseline (changes from baseline/baseline × 100) for all DXA parameters. Moreover, the relative change from baseline in total BMD was categorized in different groupings (<1/≥1%) and analyzed at week 48 using a logistic regression including analysis group, baseline value, time since menopause at screening, and BMI at baseline. Finally, a subgroup analysis was conducted for BMD L2-L4 and total BMD according to serum 25-OH D3 concentration at baseline (<75/≥75 nmol/L). The relative changes from baseline at week 48 were analyzed using ANCOVA including analysis group, baseline value, time since menopause at screening, BMI at baseline, analysis subgroup, and interaction analysis group × analysis subgroup.

For bone biomarkers, the changes from baseline in each parameter were analyzed at week 48 using a mixed model for repeated measures (MMRM), including the following covariates, in addition to analysis group and baseline value: time since menopause at screening and BMI at baseline. Comparisons within and between groups were studied. Regarding the other secondary and exploratory outcomes, depending on planned time points, an ANCOVA at week 48 including analysis group and baseline value or a MMRM including analysis group, visit, baseline values, and group*visit interaction was performed.

For all analyses, normality distribution of the residuals was verified by Skewness and Kurtosis. If the adequacy of the model could not be validated, the parameter was derived using the log transformation of the values at each time point and was then modeled with the same model characteristics. If the adequacy of the new model was not able to be validated on the log transformation, a non-parametric analysis of covariance, based on ranks (rank ANCOVA) with the same covariates was performed for the comparison between groups and Wilcoxon signed-rank test for the comparison within group.

Regarding safety, all analyses were performed on all participants who received at least one dose of study treatment according to the analysis groups. The incidence of AEs was assessed, and a description according to SOC and PT was tabulated. The number of patients with at least one TEAE was compared between analysis groups using a chi-squared test. Normal data were reported as means ± SD and non-normal data as median (Q1; Q3). All statistical tests were conducted two-sided with a significance level of 5%. No adjustment for multiplicity was considered.

## 3. Results

### 3.1. Baseline Characteristics of the Study Population

**Figure 1:**
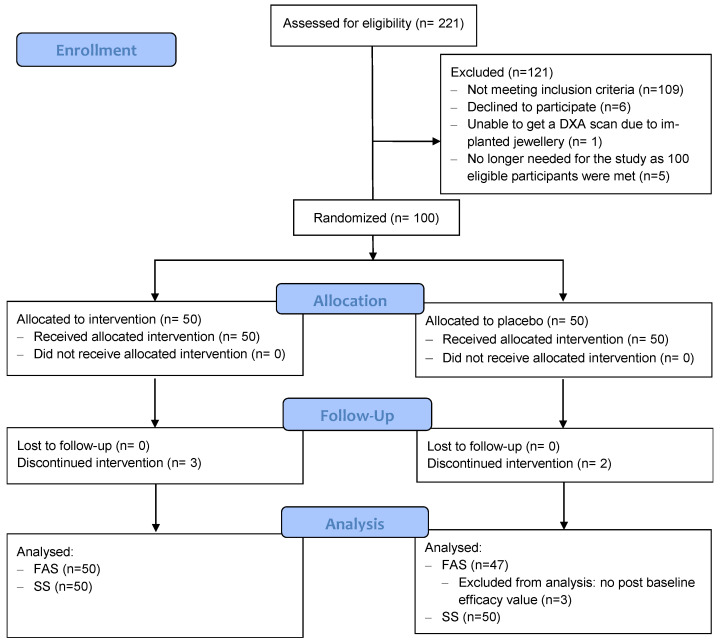
CONSORT flow diagram. FAS: full analysis set, SS: safety set.

A total of 221 women were screened, among whom 100 were deemed eligible and assigned randomly to HE (*n* = 50) or placebo (*n* = 50) groups. Five participants were lost to follow up (three in the HE group and two in the placebo group), and ninety-five participants fully completed the 48-week trial. Three participants in the placebo group were excluded from the FAS because they had no post-baseline value for any of the efficacy criteria (Figure 1).

Baseline data of the FAS population are presented in Table 1. Both groups were balanced on all the parameters presented. The participant mean age was 62.2 ± 6.3 y (range: 50; 77 y), and time since menopause was 12.6 ± 7.1 y (range: 1.1–31.8 y) with a slightly higher prevalence of women > 10 years post-menopause in the placebo group, 70% compared to 48% in the HE group (no statistical test performed). Mean BMI was 24.9 ± 3.1 (range: 18.6; −31.9), with 54% of the participants within normal range and 46% in an overweight and obese range. Mean serum 25-OH D3 concentration was 79.2 ± 27.7 nmol/L (range: 21; 155). The vitamin D status was considered sufficient if serum 25-OH D3 ≥ 75 nmol/L versus insufficient if <75 nmol/L [40]. A slightly lower prevalence of vitamin D insufficient women was observed in the HE group, 42% compared to 52% in the placebo group (no statistical test performed). All participants had osteopenia with an average T-score at the lowest site of −1.64 ± 0.41 g/cm^2^ (range: −2.4; −1.0). Two participants (*n* = 1 HE; *n* = 1 placebo) were enrolled despite that they met the exclusion criteria regarding significant endocrine disorder as they had diabetes. Their data were included in the statistical analysis as they are part of the FAS.

### 3.2. Safety and Compliance to the Intervention

Participant compliance to the IP was good with only 17% and 13% of participants who were non-compliant in the HE and placebo groups respectively. Similarly, compliance of the CaD supplements was good with only 11% and 4% of participants consuming <80% or >120% of the supplements in the HE and placebo group, respectively. Both HE and placebo capsules were well tolerated.

Analysis of the prenylflavonoids concentrations in urine and plasma confirmed the good adherence to the treatment. Indeed, X, IX, 6-PN, 8-PN, and their metabolites (glucuronide and sulfated forms) were present after 48 weeks in both urine and plasma of the HE group, while absent or negligible in the placebo group. In urine samples from the HE group, total 8-PN was detected in 94% of the participants with a mean concentration of 13.96 ± 19.11 ng/mL; total 8-PN was detected in 76% of participant plasma samples of the HE group at a mean concentration of 1.09 ± 0.92 ng/mL (Table S2).

Regarding safety, there were a total of 127 TEAEs reported during the trial, of which 55 were noted in the HE group and 77 in the placebo group (*p* = 0.21; Table S3). Among these, only 15 and 22 were suspected to be related to the IP in the HE and placebo group, respectively. Two participants in the HE group versus three in the placebo group discontinued IP and withdrew from trial due to an AE suspected to be related to the IP. Six participants experienced serious TEAEs, two occurred in the HE group and four in the placebo group. These serious TEAEs were unexpected and not related to the IP. Laboratory results were generally unremarkable (Table S4). There were no notable or clinically meaningful changes in participants’ anthropometrics or vitals detected during the study.

### 3.3. DXA Parameters

For the primary outcome, mean change in BMD at L2-L4 lumbar spine, from baseline after 48 weeks, revealed a slight but not statistically significant increase in the HE group (0.0063 ± 0.0371 g/cm^2^) compared to no change in the placebo group (0.0002 ± 0.0002 g/cm^2^). Additionally, there was no statistically significant difference detected between groups (0.0091 ± 0.0089 g/cm^2^ and 0.88 ± 0.85 in relative %).

**Figure 2:**
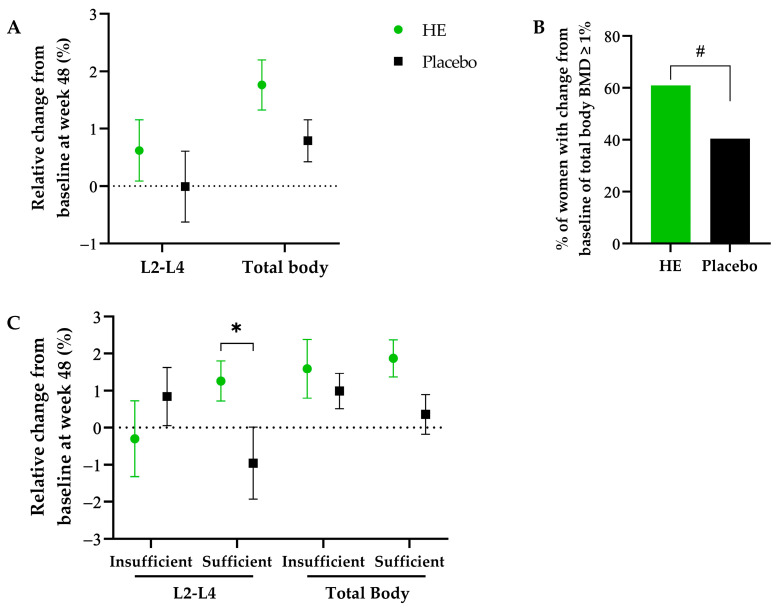
Relative changes from baseline at week 48 of BMD at L2-L4 lumbar spine and total body (**A**); percentage of women with a total body BMD change from baseline at week 48 ≥ 1% (**B**); sub-group analysis with relative changes from baseline at week 48 of BMD at L2-L4 lumbar spine and total body in vitamin D sufficient vs. insufficient women at baseline (**C**). All data are represented as mean ± SEM. # Odds ratio for relative change from baseline (probability modeled for class ≥ 1%) (HE vs. Placebo) (95% CI): 2.41 (1.01; 5.74), *p* < 0.05. * *p* = 0.05 versus placebo.

Among the other DXA parameters, there was a significant increase in the total body BMD within the HE group at week 48 compared to baseline (0.0180 ± 0.0302 g/cm^2^, *p* < 0.0001), while there was no statistically significant increase in the placebo group (0.0079 ± 0.0026 g/cm^2^). The difference between groups tended to be significant with a greater increase in the HE group compared to placebo (0.0106 ± 0.0059 g/cm^2^, *p* = 0.07; 0.99 ± 0.56 relative %, *p* = 0.08; Figure 2A). Total body BMD increased by at least 1% after 48 weeks in 61% of participants in the HE group compared to 40% in the placebo group, resulting in a significantly higher chance of having a relative change from baseline of ≥1% in the HE group versus the placebo group (adjusted odds ratio ± SE = 2.41 ± 1.07, *p* = 0.047; Figure 2B). Compared to baseline, a significant increase in BMD at femoral neck was observed in both groups after 48 weeks (0.0107 ± 0.0289 g/cm^2^ in the HE group and 0.0191 ± 0.029 g/cm^2^ in the placebo group; *p* < 0.01) without significant difference between groups.

Regarding body composition, lean mass and fat percentage were not modulated in any groups, while fat mass and visceral fat were significantly increased after 48 weeks compared to baseline in the HE group (median change (Q1; Q3) = 714.0 (−123.0; 1285.0) g for fat mass and 52.5 (−55.0; 213.0) g for visceral fat, *p* < 0.05), but no significant difference between groups was found.

Additionally, post hoc sub-group analysis was performed according to vitamin D status (sufficient if ≥75 nmol/L and insufficient if <75 nmol/L). In vitamin D sufficient women, there was an increase in the HE group compared to the placebo group for the BMD at L2-L4 lumbar spine (difference of adjusted relative changes from baseline ± SE = 2.29 ± 1.16%; *p* = 0.051) and the total body BMD (difference of adjusted relative changes from baseline ± SE = 1.44 ± 0.78%, *p* = 0.066; Figure 2C).

### 3.4. Biochemical Analysis

No significant differences were observed between the HE and placebo groups after 48 weeks of supplementation for any of the plasma bone biomarkers measured (Table S5). Compared to baseline, CTx level increased after 48 weeks in both groups (*p* < 0.001), while sclerostin and TRAP5b decreased in both groups (*p* < 0.05). uOC decreased significantly in the HE group after 48 weeks (*p* < 0.01).

Similarly, no significant differences between groups were observed at 48 weeks for the following blood parameters: triglycerides, total cholesterol, HDL-cholesterol, LDL-cholesterol, fasting glucose, insulinemia, HbA1c, HOMA-IR, serum 25-OH D3 concentration, and 17-β oestradiol (Table S6).

### 3.5. Antropometrics, Physical Activity, Dietary Intake and Health-Related Quality of Life

No significant differences between groups were observed for anthropometric parameters at any visit (Table S6). Physical activity assessed using the PASE questionnaire showed similar activity at baseline and throughout the 48 weeks between the HE and placebo groups (Table S7). 

Dietary analysis using the FFQ showed both groups had similar intake at baseline. After 48 weeks, the HE group showed higher fat, calcium, and vitamin K_2_ intakes, compared to the placebo group (*p* < 0.05; Table S7). For fat, the median change from placebo at week 48 was + 11 g/d, for calcium +112 mg/d, and for vitamin K_2_ + 2.3 µg/d in the HE group compared to the placebo group.

Changes in SF-36 scores after 48 weeks are shown in Table 2. The physical functioning score was significantly increased in the HE group compared to the placebo group (*p* < 0.05), with 25 participants (53%) showing increased scores (>0) in the HE group compared to 14 (30%) in the placebo group. The role limitations due to physical health score trended toward a greater increase in the HE group compared to placebo (*p* = 0.08); however, at least 50% of the participants had no change in both groups (Q1; Q3 changes from baseline = 0; 0), and only eight and five participants had increased scores in the HE and placebo group, respectively, indicating a weak effect. No difference between groups was observed for the other scores.

### 3.6. Gut Microbiome Modulation

**Figure 3:**
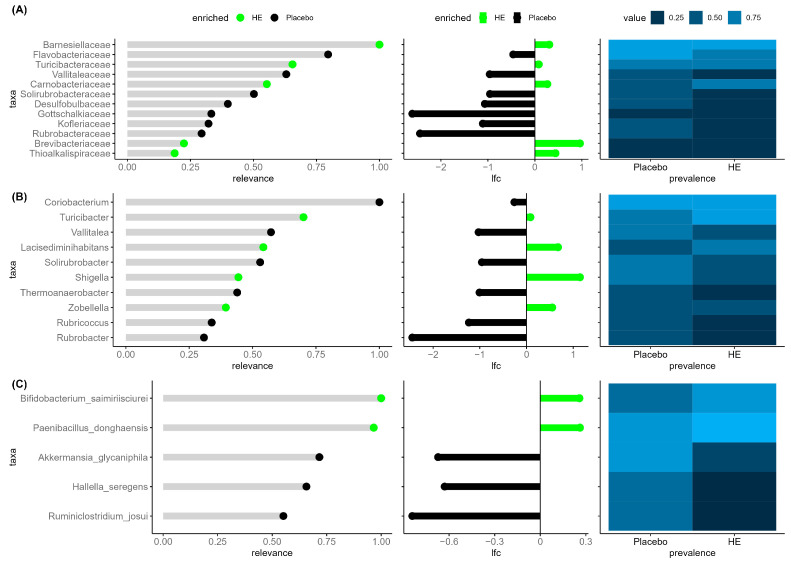
Microbiome components differentiating between HE and placebo at week 48. For each specific level ((**A**). Family, (**B**). Genus and (**C**). Species), we displayed in the left panel the importance of each selected taxon to differentiate between the two groups, in the middle panel the effect size, by reporting the log-fold difference between the mean abundance of the taxon in each group, and finally in the rightmost panel the prevalence of the selected taxa on each compared group. Green = HE; Black = placebo.

As the gut microbiota is a key player in prenylflavonoid metabolism and bone homeostasis, potential differences in the microbiome composition between the HE and placebo groups were explored. Low-dimensional representations of the taxonomic profiles computed using non-metric multidimensional scaling (MDS) on Bray–Curtis and Jaccard β-diversity scores suggested that there were no differences between the groups at any visit. These exploratory results were also confirmed with MiRKAT overall association tests based on β-diversity (Figure S1). There was also no significant difference between groups in terms of α-diversity assessed as change from baseline by the inverse Simpson index, Shannon index, and observed number of species (Figure S2). Despite this lack of significant differences between the overall compositions of samples between the HE and placebo groups, an exploratory multivariate analysis was run to identify the taxa that seem more relevant to distinguish between the two groups. Results for each taxonomic rank (family, genus, specie) are displayed in Figure 3.

Results shown in Figure 3A highlight five families enriched in the HE group and seven enriched in the placebo group. The most discriminant family to differentiate between the two groups was *Barnesiellaceae,* which was more abundant in the HE group. Observed prevalence, however, was high and roughly the same in both groups. *Turicibacteraceae* was also enriched in the HE group, showing larger mean abundance and prevalence than in the placebo group. This finding was consistent with the identification of *Turicibacter* as a differentiating genus more abundant and prevalent in the HE group. To be noted, *Turicibacter* was also identified as one of the most discriminant genus to differentiate between the two groups after 24 weeks, being more abundant and prevalent in the HE group compared to the placebo group such as after 48 weeks (Figure S3). *Shigella* was also found more abundant in the HE group after 48 weeks. These two genera were largely the most abundant among the selected most discriminant genera. Two other genera were more abundant in the HE group, and six others were more abundant in the placebo group with *Coriobacterium* being the most relevant. Among enriched species identified in the HE group, *Bifidobacterium saimiriisciurei* and *Paenibacillus donghaensis* had both larger mean abundance and prevalence in the HE group compared to the placebo group. *Akkermansia glycaniphila*, *Hallella seregens,* and *Ruminiclostridium josui* were more abundant and prevalent in the placebo group. 

Finally, no difference between groups was observed regarding *E. limosum* abundance at baseline and after 48 weeks of supplementation. We further explored if treatment responsiveness (total body BMD) was correlated with relative abundance of *E. limosum* at baseline. There was no specific pattern relating responsiveness to the observed abundance (Figure S4). 

Changes at week 24 and 48 in the profile of total and individual SCFA were not significantly different between groups (Table S8).

## 4. Discussion

To the best of our knowledge, this is the first randomized controlled trial (RCT) conducted to examine the effect of a hop extract on bone health in postmenopausal women with osteopenia. In this population, we demonstrated that a daily supplementation with 100 µg of 8-PN from a standardized hop extract for 48 weeks increased total body BMD compared to placebo. An improvement of the SF-36 physical functioning score was also observed in the HE group suggesting a higher perceived ability to perform daily activities. However, no significant effect was found in BMD at specific sites (lumbar spine, femoral neck, and total hip), plasma bone biomarkers, and other secondary outcomes, notably blood lipids and glucose homeostasis parameters.

This beneficial impact on BMD is consistent with previous studies performed in the ovariectomized rat with standardized hop extract, which showed an increase in BMD following 8 to 12 weeks of supplementation while using a daily dose of 8-PN from 6 to 27 times higher in a human dose equivalent [13,14]. Regarding the effects of other types of phytoestrogens in humans, isoflavones are the most studied in terms of bone health effect in postmenopausal women. The recent meta-analysis by Sansai et al. included 63 RCTs and various types of isoflavone interventions [8]. A statistically significant increase in BMD was found at the lumbar spine, femoral neck, and distal radius with isoflavones. However, these favorable effects were predominantly associated with the use of genistein pure compounds and synthetic isoflavones, while the benefits of isoflavone extracts and dietary isoflavone supplements on BMD remain inconclusive (with up to 300 mg/day of isoflavone aglycone equivalents). This analysis did not reveal any significant effects on the total hip and the total body, which might be because no study has investigated the effects of genistein or synthetic isoflavones on those two sites.

Bone turnover biochemical markers help clinicians to identify patients at high risk for fracture and to monitor the efficacy of osteoporosis treatments. However, no significant difference between the HE and placebo groups was observed regarding the bone biochemical markers concentrations after 48 weeks of supplementation. Among the available biochemical markers, pro-collagen type I *N-*terminal propeptide (PINP) and *C-*terminal telopeptide (CTX) have been recommended as reference biochemical markers of bone formation and bone resorption, respectively [41]. Surprisingly, CTX increased compared to baseline in both groups, while PINP remained stable. The marker of bone resorption TRAP5b and sclerostin, a marker known to increase in bone diseases, were both decreased in the HE and placebo groups compared to baseline. Finally, a decrease in serum undercarboxylated osteocalcin (uOC) was observed only in the HE group compared to baseline, which could be a sign of a beneficial impact on BMD in the HE group. Indeed, uOC concentration is a marker of bone turnover, increased serum uOC levels have been associated with an increased risk of hip fracture [42] and of low BMD of the hip and spine in postmenopausal women [43].

The magnitude of HE effects on bone density is not comparable to those of osteoporosis medications, but it could be of interest as a preventive measure for women with low bone mass that cannot be prescribed medication at this stage. Dawson-Hughes et al. have reported that after 3 years of vitamin D (700 IU) and calcium (500 mg) supplementation, a 1.1% net increase in total body BMD compared to placebo was associated with a relative risk reduction of 0.4 of first osteoporotic fracture in men and women [44]. Accordingly, in our study, the mean total body BMD increase of 1% and the significantly higher proportion of women with an increase ≥1% following HE consumption compared to placebo might point toward a similar relative risk reduction. Notably, considering that this 1% net increase is in addition to a 0.8% increase already observed in both groups in the context of CaD supplementation. Moreover, several studies have found reduced muscle strength, reduced physical capacity, and reduced quality of life among patients with low BMD [45,46,47]. Notably, in a cross-sectional study, 18 postmenopausal women with osteopenia and a healed wrist fracture had a lower score in the sub-scales on the SF-36 quality of life questionnaire for physical functioning, role limitation due to physical problem, bodily pain and vitality compared to a matched, healthy control group with no previous fracture [47]. Therefore, the increase in the physical functioning SF-36 sub-scale following HE supplementation compared to placebo may indicate an improved quality of life in women with osteopenia.

Dawnson Hugues et al. have reported in older women without intervention an annual bone loss of about 1% in the total body and 0.8% in the lumbar spine region [44]. Furthermore, inadequate intakes of vitamin D and calcium lead to increased bone loss, and CaD supplementation has been demonstrated to be effective in reducing bone loss in postmenopausal women [48]. In our study, all women were supplemented with CaD, which placed favorable conditions of bone loss prevention, therefore potentially limiting the ability to detect an effect of the HE alone. In the placebo group, supplementation with 1000 mg of calcium and 800 UI of vitamin D resulted in a mean net increase of 0.8% at the total body, 2.2% at the femoral neck, and no change at the lumbar spine observed after 48 weeks. Similar total body BMD increase has been previously reported with CaD supplementation [49,50,51,52]. Notably, an open-label, randomized, controlled trial investigated in 590 postmenopausal women the effect of a similar CaD supplementation or no intervention over 3 years [50]. A significant increase in total body BMD of 0.8% was observed in the intervention group compared to control group (0.2%), but no significant differences were observed at the lumbar spine, femoral neck, trochanter, and total proximal. In compliant women (those who took at least 80% of their supplementation), greater effect was observed with a significant increase in BMD at the total body (1.3%, i.e., around 0.4% per year) and all specific sites investigated. Interestingly, these women had a mean baseline serum 25OHD level of 50 nmol/L, which suggests that they were moderately vitamin D insufficient. It is known that vitamin insufficient women benefit more from a higher CaD intake [53]. In our study, following a subgroup analysis, according to vitamin D status, we observed that vitamin D-insufficient women (defined here as <75 nmol/L) might have benefited more from the CaD supplements, as in the placebo group, an increase of 0.8% was observed in these women, while a decrease of 1% was observed in vitamin D-sufficient women. Moreover, we observed that vitamin D-sufficient women seemed to have a more beneficial impact of HE supplementation compared to vitamin D-insufficient women, as an increase of the lumbar spine BMD (+2.3% HE vs. placebo) was observed primarily in this vitamin D-sufficient population.

The main mechanism of action of HE is the estrogenic activity of 8-PN, which has been demonstrated in vitro and in vivo and which has been recently extensively reviewed [9,10,11,54]. Along with its metabolites, 8-PN was only detected in the plasma and urine of women supplemented with HE, confirming adherence to treatment and an exposition to these active compounds in this group only. No correlation was found between the levels of total 8-PN and change in total body BMD after 48 weeks. However, the samples were collected 24 h after the last capsule ingestion and therefore are not representative of the acute levels of metabolites circulating after HE consumption. Another potential mechanism of action could be the antioxidant properties of the standardized hop extract. Indeed, changes in reactive oxygen species (ROS) and/or antioxidant systems seem to be involved in the pathogenesis of bone loss. Additionally, a marked decrease in plasma antioxidants was found in osteoporotic women, and recent data suggest that diet supplementation with antioxidants could be an effective strategy to prevent bone loss [55]. Hops prenylflavonoids, and particularly xanthohumol (X), are known to exert diverse antioxidant and free-radical-scavenging properties and therefore might have beneficially impacted BMD of postmenopausal women supplemented with HE [56].

Despite the fact that volunteers were asked to maintain a similar diet throughout the study, significantly higher changes from baseline at week 48 were observed for fat, vitamin K_2_, and calcium in the HE group compared to the placebo group. No difference was observed between groups in terms of weight, fat mass, and blood lipids, suggesting that the small difference of fat intake (around 11 g) had no impact on these parameters. The minimum efficacy dose of vitamin K_2_ for osteopenia and osteoporosis is known to be of 45 mg/d; therefore, the differential amount of +2.3 µg/d (20,000 times less) observed here is unlikely to be responsible for the effect on BMD [57]. Finally, the difference of calcium between groups does not take into account the calcium supplementation, i.e., an additional 1000 mg/d of calcium in all women in addition to the initial reported mean intake of 1170 mg/d at baseline. Therefore, it is also unlikely that the differential amount of calcium of +112 mg/d could have contributed to the observed effect. 

Another hypothesis for the important variability in response observed in this trial could be the inter-individual variation in prenylflavonoids metabolism that was reported previously [20,21,22]. The final level of 8-PN absorbed does not only depend on the presence of 8-PN itself in the HE but also on the transformation of its precursor IX into 8-PN by the intestinal bacteria *E. limosum* [21]. However, important inter-individual variability was observed for conversion efficacy with reported low, moderate, and high 8-PN producers in humans [19]. *E. limosum* levels were not statistically different between the HE and placebo group at baseline and after 48 weeks. Furthermore, subset analysis based on responsiveness to HE supplementation did not indicate any difference in *E. limosum* levels at baseline and after 48 weeks between the women with an increase in their total body BMD ≥1% vs. <1%. Likewise, the 8-PN levels detected in blood or urine in women who took the HE supplementation were not correlated with the magnitude of responsiveness. Altogether, these results suggest that conversion capability of the women included in this trial was not the primary cause of efficacy. Furthermore, it is likely that a plateau effect was reached with the dose of 100 µg of 8-PN and that any additional amount brought by the conversion was not responsible for additional bone health effect. Interestingly, in the study conducted by Heyerick et al., using the same extract, 100 µg of 8-PN was sufficient to relieve women from postmenopausal symptoms, and no additional benefit was observed with the higher dosage of 250 µg of 8-PN [17]. 

A novel aspect of this trial was the analysis of the participants’ gut microbiome and its relationship to the responsiveness of treatment. No difference between HE and placebo was observed for overall association parameters, alpha-diversity indices, and SCFA levels throughout the study, hence indicating no major shift in the gut microbiome composition and function. However, the explorative multivariate analysis indicated that after 48 weeks among the taxa identified as the most relevant to discriminate the two groups, there was notably a higher abundance of the genera *Turicibacter* and *Escherichia Shigella* in the HE group. In a recent animal study, positive correlations between the genera *Turicibacter* and total BMD were observed in ovariectomized mice supplemented with new antidepressant drug (R)-ketamine [58]. (R)-ketamine significantly attenuated the reduced abundance of *Turicibacter* observed after ovariectomy compared to sham control. Moreover, *Escherichia Shigella* was found to be more abundant in individuals with osteopenia compared to those with osteoporosis in a cohort of 181 older adults (including 150 women) [59]. Conversely, with the latter study and our results, *Escherichia Shigella* was also found negatively associated with L1-L4 BMD, total BMD, and femur total BMD in the UK biobank cohort study including middle-aged adults [60]. These results suggest that there may be an association between *Escherichia Shigella* and BMD modulation, but causal relationship needs to be further investigated. Other species identified as discriminating the two groups have not yet been related to bone health and disease.

Strengths of this study include the sample size calculation, the robust design (randomized, placebo-controlled, double-blind), the good adherence rate, the low drop-out rate, and the integrated analysis, including the impact of treatment on the microbiome and HE metabolites. The main limitation is the relatively short duration considering bone loss, as a duration of at least 2 years would incorporate more complete bone remodeling cycles and further strengthen the evidence provided by DXA in this trial. However, the bone remodelling cycle is known to last 120–200 days [61]; therefore, a duration of 48 weeks has allowed 1.5 to 2 bone remodelling cycles to happen. 

To conclude, in postmenopausal women with osteopenia, daily consumption of a standardized hop extract with 100 µg of 8-PN during 48 weeks was found to have a beneficial increase of 1% of the total body BMD compared to placebo, above and beyond an increase associated with a calcium and vitamin D supplementation. Furthermore, even if no major shift of the gut microbiota composition was observed, modulation of some taxa previously identified as associated with bone loss was noted in the HE group and deserves further investigation. New clinical trials with notably a longer duration are needed to confirm the beneficial effect of standardized hop extract on bone health.

## Figures and Tables

**Table 1:** Baseline characteristics of the HE and placebo group.

Parameter	Unit	HE (*n* = 50)	Placebo (*n* = 47)
Age	years	60.8 (6.3)	63.6 (6.1)
Time since last menstruation	years	11.1 (7.3)	14.1 (6.6)
Anthropometrics			
Weight	kg	64.4 (9.0)	67.9 (7.5)
BMI	kg/m^2^	24.5 (3.2)	25.3 (3.0)
Waist circumference	cm	81.4 (7.8)	84.0 (7.9)
Hip circumference	cm	100.1 (6.9)	101.9 (6.8)
Waist-to-hip ratio	a.u.	0.8 (0.1)	0.8 (0.1)
Lifestyle			
Energy intake	Kcal/day	2044.6 (539.5)	1960.5 (570.8)
Calcium intake	mg/day	1201.8 (364.1)	1140.4 (432.0)
Vitamin D intake	µg/day	4.87 (3.31)	4.71 (2.76)
Physical activity, PASE total score	a.u.	165.0 (62.2)	185.9 (67.6)
DXA parameters			
BMD at L2-L4	g/cm^2^	1.02 (0.07)	1.04 (0.10)
BMD at FN	g/cm^2^	0.85 (0.09)	0.84 (0.07)
BMD total body	g/cm^2^	1.06 (0.07)	1.07 (0.08)
T-score at L2-L4	a.u.	−1.50 (0.59)	−1.30 (0.86)
T-score at FN	a.u.	−1.09 (0.73)	−1.19 (0.59)
Lowest T-score at inclusion	a.u.	−1.64 (0.41)	−1.64 (0.41)
Lean mass	kg	38.0 (3.7)	39.7 (3.5)
Fat mass ^1^	kg	23.5 (6.9)	26.0 (8.9)
Visceral fat	kg	1.9 (0.8)	2.2 (0.8)
% Fat	%	37.4 (6.6)	38.4 (6.5)
FRAX—Major osteoporotic	%	8.14 (5.18)	9.81 (4.98)
FRAX—Hip fracture	%	1.68 (3.07)	1.79 (1.40)
Blood parameters			
25-OH D3	nmol/L	83.4 (31.2)	74.7 (22.8)
Calcium	mmol/L	2.43 (0.10)	2.39 (0.09)
17-β estradiol	pmol/L	20.7 (7.5)	18.5 (0.0)
Glucose	mmol/L	4.70 (0.48)	4.73 (0.45)
Insulin	mIU/L	5.1 (2.3)	5.0 (2.4)
HbA1c	mmol/L	35.4 (3.4)	36.2 (2.7)
Total cholesterol	mmol/L	5.7 (1.0)	5.7 (1.0)
HDL cholesterol	mmol/L	1.9 (0.4)	1.7 (0.3)
LDL cholesterol	mmol/L	3.4 (1.2)	3.6 (1.0)
Triglycerides	mmol/L	0.92 (0.46)	1.12 (0.65)
HOMA IR	a.u.	1.08 (0.58)	1.06 (0.57)

^1^ One aberrant data has been removed after statistical analysis for this parameter. Mean (SD).

**Table 2:** Health-related quality of life.

SF-36 Scores	Statistics	HE (*n* = 50)	Placebo (*n* = 47)	*p*
Physical functioning	median (Q1; Q3)	5 (0; 10)	0 (0; 5)	0.049
Role limitations due to physical health	median (Q1; Q3)	0 (0; 0)	0 (0; 0)	0.082
Role limitations due to emotional problems	median (Q1; Q3)	0 (0; 0)	0 (0; 0)	0.589
Energy/fatigue	mean (SD)	2.66 (11.7)	1.6 (12.69)	0.914
Emotional well-being	median (Q1; Q3)	0 (−4; 8)	0 (−4; 4)	0.530
Social functioning	median (Q1; Q3)	0 (0; 0)	0 (0; 0)	0.555
Pain	mean (SD)	1.01 (18.96)	1.06 (23.06)	0.969
General health	mean (SD)	2.77 (10.67)	−0.43 (14.74)	0.407

## Data Availability

The data presented in this study are available on request from the corresponding author.

## Supplementary Materials

The following supporting information can be downloaded at: https://www.mdpi.com/article/10.3390/nu15122688/s1, Figure S1: β-diversity at baseline (V2), 24 weeks (V4), and 48 weeks (V6) in the HE and placebo group; Figure S2: Evolution of α-diversity as change from baseline after 24 weeks (V4) and 48 weeks (V6) in the HE and placebo group; Figure S3: Microbiome components differentiating between HE and placebo at week 24; Figure S4: *Eubacterium limosum* relative abundance between treatment group (A) and between responders and non-responders (B); Table S1: Targeted compounds and performance of the LC-MS method; Table S2: Plasma and urine prenylflavonoids and their metabolites; Table S3: Overview of adverse events on all randomized participants; Table S4: Blood safety parameters at baseline and 48 weeks; Table S5: Plasma bone biomarkers at 48 weeks; Table S6: Anthropometrics and blood efficacy parameters at 48 weeks; Table S7: Dietary intake and physical activity at 48 weeks; Table S8: SCFA concentrations at 24 wand 48 weeks.
