# Supplementary material for: Effect of a Hop Extract Standardized in 8-Prenylnaringenin on Bone Health and Gut Microbiome in Postmenopausal Women with Osteopenia: A One-Year Randomized, Double-Blind, Placebo-Controlled Trial
Source: Nutrients. 2023 Jun 9;15(12):2688. doi: 10.3390/nu15122688 (PMC10304064; doi:10.3390/nu15122688)
Supplement: Supplementary file 1 [file nutrients-15-02688-s001.zip › nutrients-2406034-supplementary.pdf]

Supplementary material

Effect of a Hop Extract Standardized in 8-Prenylnaringenin on Bone Health and Gut Microbiome in Postmenopausal Women with Osteopenia: A One-Year Randomized, Double-Blind, Placebo-Controlled Trial

(A)

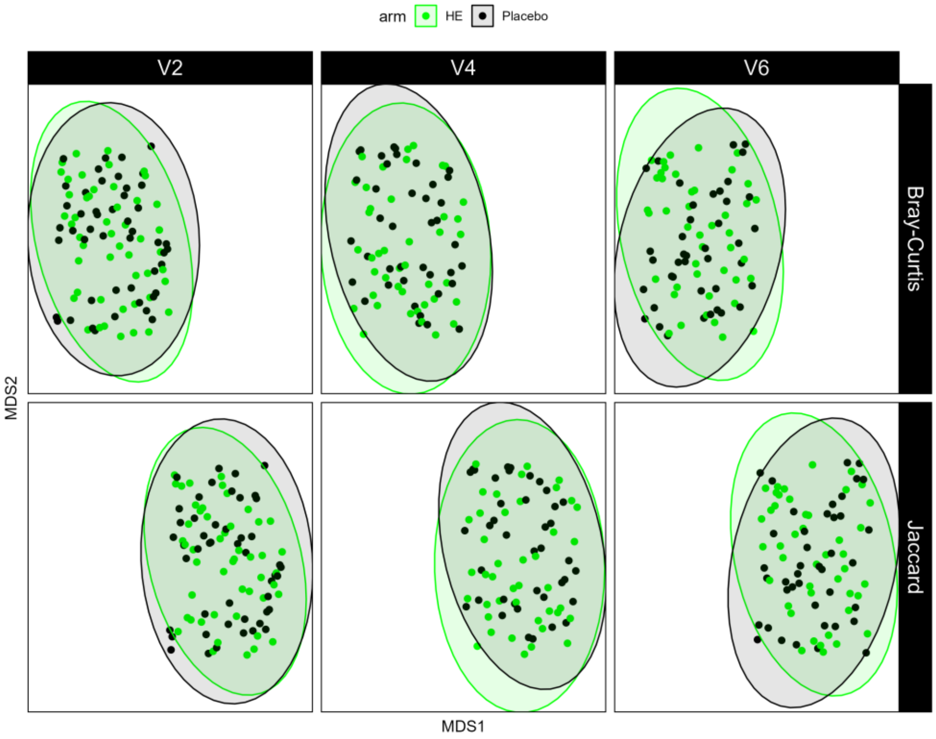

(B)

Visit	Rank	Bray-Curtis	Jaccard
V2	Family	0.34	0.38
	Genus	0.37	0.43
	Species	0.49	0.55
V4	Family	0.44	0.44
	Genus	0.50	0.54
	Species	0.57	0.56
V6	Family	0.51	0.51
	Genus	0.55	0.52
	Species	0.29	0.27

Figure S1. β-diversity at baseline (V2), 24 weeks (V4), and 48 weeks (V6) in the HE and placebo group assessed by (A) low-dimensional representations of the taxonomic profiles computed using non-metric multidimensional scaling (MDS) on Bray-Curtis and Jaccard pairwise dissimilarities and (B) associated p-values from MiRKAT tests of association with treatment groups.

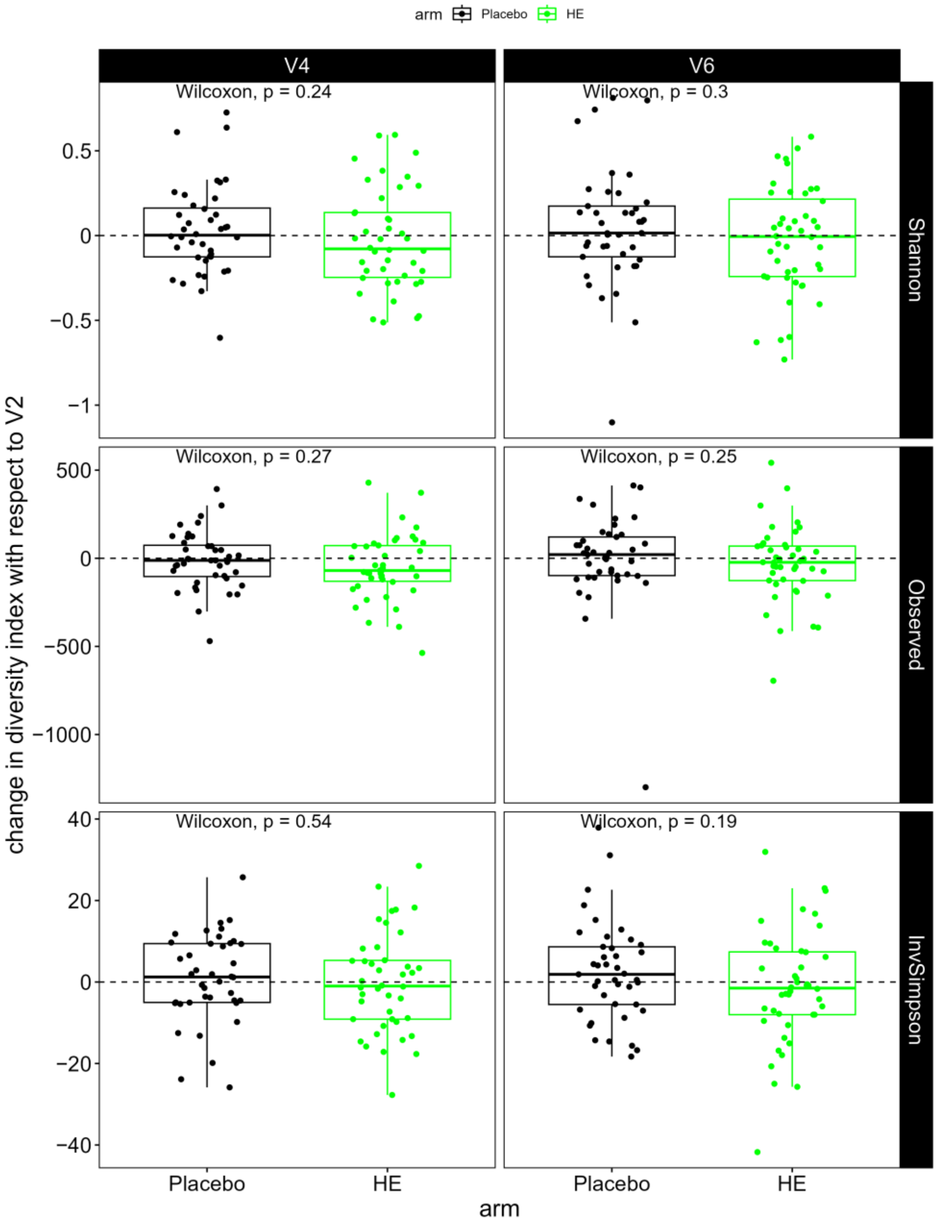

Figure S2. Evolution of α-diversity as change from baseline after 24 weeks (V4) and 48 weeks (V6) in the HE and placebo group assessed by the Shannon index, observed number of species (Observed), and the inverse Simpson index (InvSimpson). Reported p-values are from two-sample Wilcoxon rank sum tests.

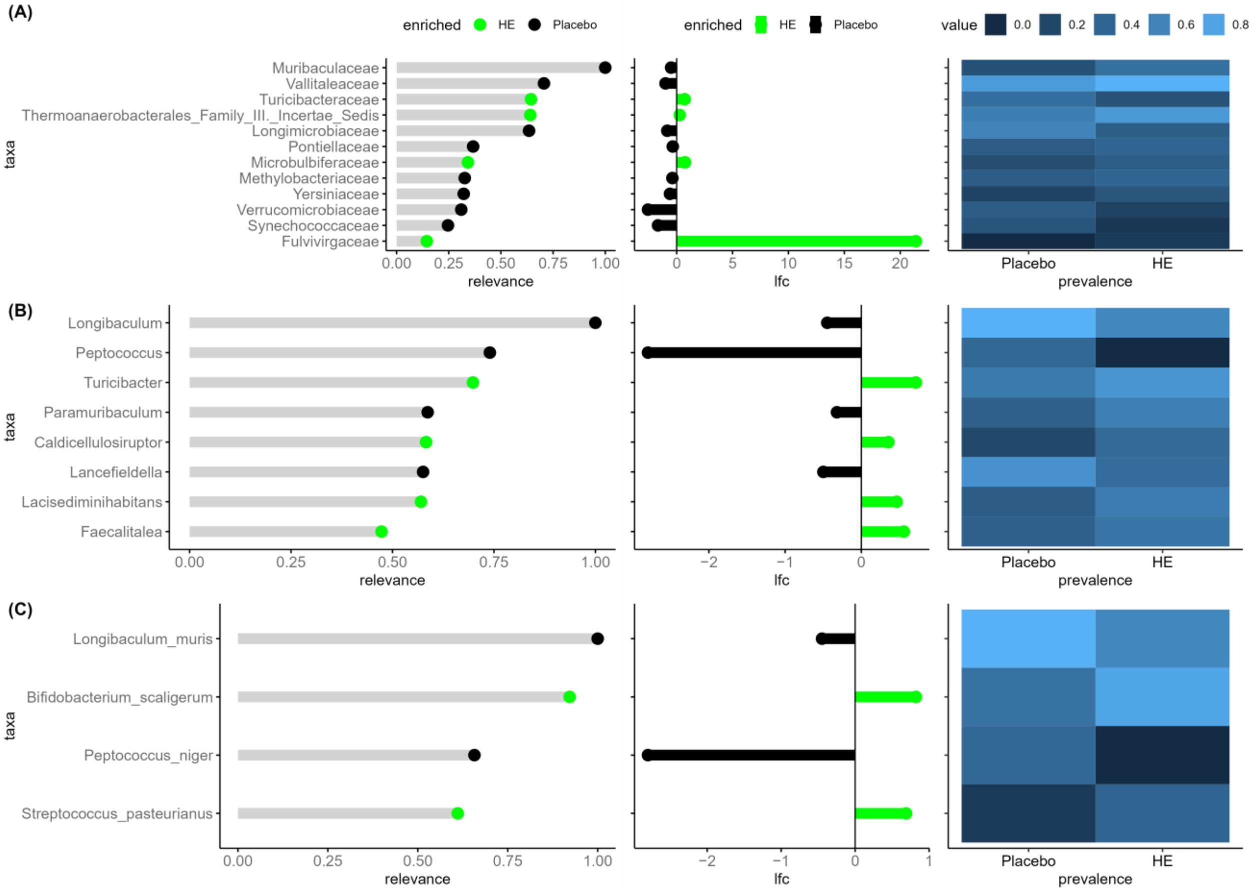

Figure S3. Microbiome components differentiating between HE and placebo at week 24. For each specific level (**A**. Family, **B**. Genus and **C**. Species) we displayed in the left panel the importance of each selected taxon to differentiate between the two groups, in the middle panel the effect size, by reporting the log-fold difference between the mean abundance of the taxon in each group and finally in the rightmost panel the prevalence of the selected taxa on each compared group.

(A)

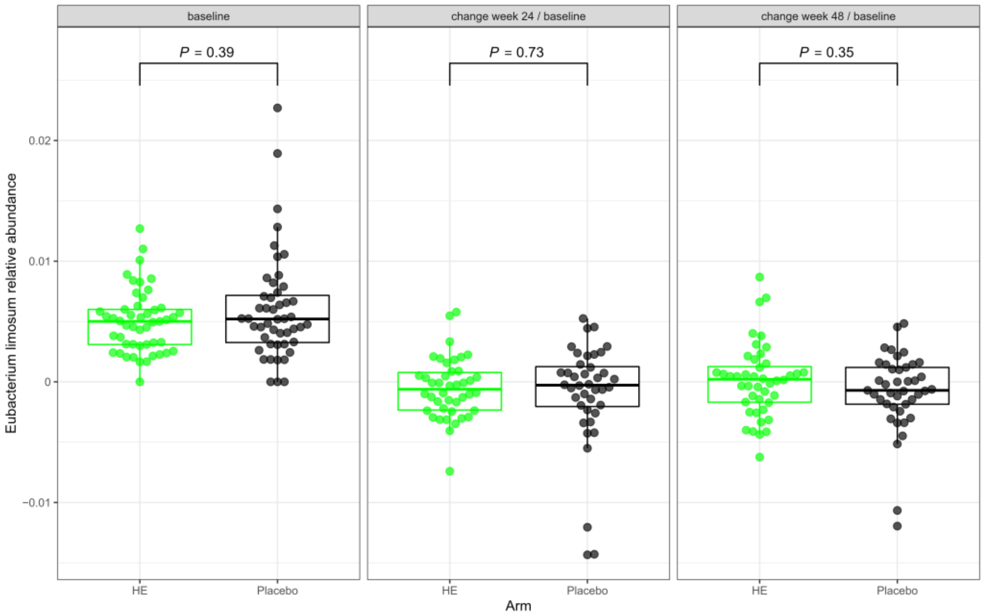

(B)

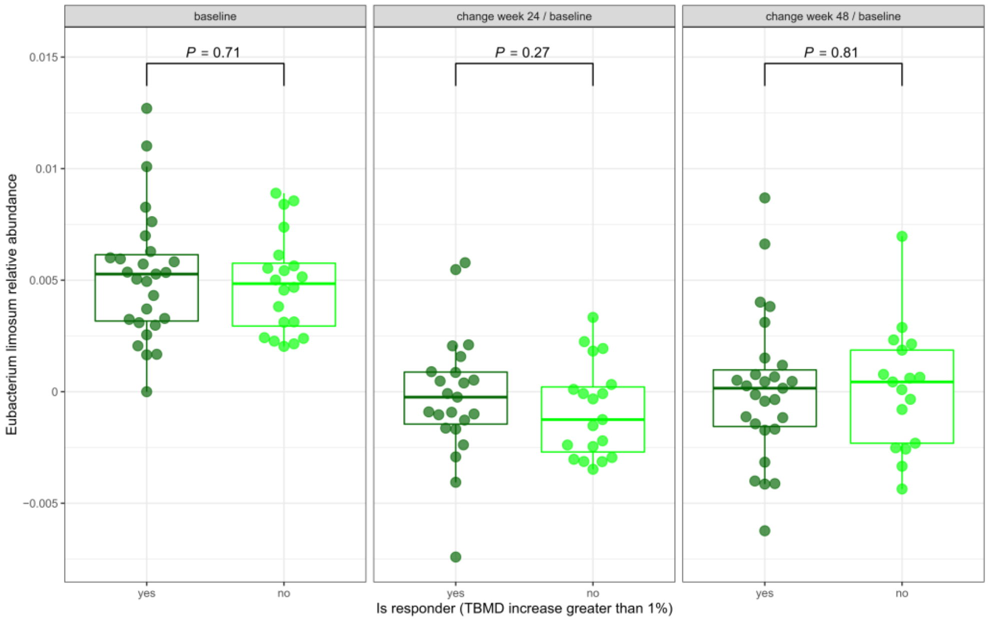

Figure S4. *E. limosum* relative abundance between treatment group (A) and between responders and non-responders (B). Responders are defined as women in the HE group with a total body BMD (TBMD) relative change from baseline at week 48 ≥ 1%.

Table S1. Targeted compounds and performance of the LC-MS method: Retention Time, m/z, repeatibility, linearity and limit of quantification (LOQ.).

Compound	Formula	Retention Time (min)	[M – H]−	RSD % in Diluent	RSD % in Spiked Plasma	Linearity (R^2)	LOQ (ng/mL)
IX	$C_{21}H_{22}O_5$	6.6	353.13944	3.2	3.8	0.9996	0.14
8-PN	$C_{20}H_{20}O_5$	7.4	339.12379	3.5	2.5	0.9997	0.11
6-PN	$C_{20}H_{20}O_5$	8.2	339.12379	4.5	2.7	0.9997	0.1
X	$C_{21}H_{22}O_5$	8.5	353.13944	4.7	3.5	0.9999	0.11

Table S2. Plasma and urine prenylflavonoids and their metabolites.

		8-PN Total (ng/mL)		6-PN Total (ng/mL)		X Total (ng/mL)		IX Total (ng/mL)	
	**Statistic**	**0**	**48**	**0**	**48**	**0**	**48**	**0**	**48**
**Plasma**									
HE (n = 50)	n	0	38	0	1	0	30	0	46
	%	0.0	76.0	0.0	2.0	0.0	60.0	0.0	92.0
	Median (Q1; Q3)	NA	0.50 (0.50; 1.21)	NA	0.50 (0.50; 0.50)	NA	0.50 (0.50; 0.50)	NA	4.16 (2.58; 8.94)
Placebo (n = 47)	%	0.0	1	0.0	2.0	0.0	60.0	0.0	92.0
	%	0.0	2.1	0.0	0.0	0.0	6.4	0.0	0.0
	Median (Q1; Q3)	NA	0.50 (0.50; 0.50)	NA	NA	NA	0.50 (0.50; 0.50)	NA	NA
**Urine**									
HE (n = 50)	n	4	47	1	46	0	47	4	47
	%	8	94	2	92	0	94	8	94
	Median (Q1; Q3)	NA	6.67 (4.16; 15.46)	NA	2.46 (1.55; 3.86)	NA	1.31 (0.81; 2.56)	NA	86.30 (48.59; 131.66)
Placebo (n = 47)	n	1	0	0	0	0	0	0	1
	%	2.1	0.0	0.0	0.0	0.0	0.0	0.0	2.1
	Median (Q1; Q3)	NA	NA	NA	NA	NA	NA	NA	3.80 (3.80; 3.80)

Table S3. Overview of adverse events on all randomized participants.

Parameter, Units of Measure		HE (n = 50)	Placebo (n = 50)
Number of AEs, n		61	77
Number of TEAEs, n	Total *	55	72
	Mild	39	55
	Moderate	14	13
	Severe	2	4
Number of subjects with TEAEs, n (%)		30 (60.0)	36 (72.0)
Number of subjects with serious TEAEs, n (%)		2 (4.0)	2 (4.0)
Number of subjects with severe TEAEs, n (%)		2 (4.0)	2 (4.0)
Number of subjects with mild or moderate TEAEs, n (%)		35 (70.0)	42 (84.0)
Number of subjects with treatment related or suspected TEAEs, n (%)		15 (30.0)	22 (44.0)

AE, Adverse Event; TEAE, Treatment Emergent Adverse Event; HE, Hop Extract

* Chi-square test: p-value = 0.2053

Table S4. Blood safety parameters at baseline and 48 weeks.

Parameters	Time	Statistics	HE (N = 47)	Placebo (N = 47)
**Hematology**				
White Blood Cells (10^9/L)	Baseline	Mean (SD)	4.855 (1.184)	5.734 (1.634)
	Week 48	Mean (SD)	5.060 (1.205)	5.857 (2.002)
		Low n (%)	9 (19.1%)	3 (6.4%)
		Normal n (%)	38 (80.9%)	42 (89.4%)
		High n (%)	0	2 (4.3%)
Red Blood Cells (10^{12}/L)	Baseline	Mean (SD)	4.419 (0.274)	4.427 (0.247)
	Week 48	Mean (SD)	4.461 (0.269)	4.512 (0.273)
		Normal n (%)	46 (97.9%)	45 (95.7%)
		High n (%)	1 (2.1%)	2 (4.3%)
Haemoglobin (g/dL)	Baseline	Mean (SD)	13.40 (0.84)	13.34 (0.75)
	Week 48	Mean (SD)	13.41 (0.81)	13.41 (0.87)
		Low n (%)	22 (46.8%)	22 (46.8%)
		Normal n (%)	25 (53.2%)	23 (48.9%)
		High n (%)	0	2 (4.3%)
Haematocrit (L/L)	Baseline	Mean (SD)	0.3997 (0.0224)	0.4001 (0.0204)
	Week 48	Mean (SD)	0.4066 (0.0208)	0.4101 (0.0243)
		Normal n (%)	47 (100.0%)	45 (95.7%)
		High n (%)	0	2 (4.3%)
Mean Corpuscular Volume (fL)	Baseline	Mean (SD)	90.55 (3.79)	90.44 (3.04)
	Week 48	Mean (SD)	91.26 (3.98)	90.96 (3.28)
		Low n (%)	1 (2.1%)	0
		Normal n (%)	44 (93.6%)	46 (97.9%)
		High n (%)	2 (4.3%)	1 (2.1%)
Mean Corpuscular Haemoglobin (pg)	Baseline	Mean (SD)	30.36 (1.45)	30.14 (0.99)
	Week 48	Mean (SD)	30.19 (1.53)	29.40 (2.45)
		Low n (%)	3 (6.4%)	5 (10.6%)
		Normal n (%)	43 (91.5%)	42 (89.4%)
		High n (%)	1 (2.1%)	0
Mean Corpuscular Haemoglobin Concentration (g/dL)	Baseline	Mean (SD)	33.53 (0.83)	33.34 (0.87)
	Week 48	Mean (SD)	32.99 (0.83)	32.70 (0.77)
		Low n (%)	4 (8.5%)	10 (21.3%)
		Normal n (%)	43 (91.5%)	37 (78.7%)
Red Cell Distribution Width (%)	Baseline	Mean (SD)	13.07 (0.61)	13.06 (0.53)
	Week 48	Mean (SD)	13.13 (0.74)	12.92 (2.43)
		Low n (%)	0	2 (4.3%)
		Normal n (%)	45 (95.7%)	42 (89.4%)
		High n (%)	2 (4.3%)	3 (6.4%)
Platelets (10^9/L)	Baseline	Mean (SD)	270.7 (52.6)	260.7 (48.9)
	Week 48	Mean (SD)	272.6 (53.1)	269.4 (45.0)
		Low n (%)	1 (2.1%)	0
		Normal n (%)	44 (93.6%)	47 (100.0%)
		High n (%)	2 (4.3%)	0
Plateletcrit (%)	Baseline	Mean (SD)	0.272 (0.047)	5.641 (36.865)
	Week 48	Mean (SD)	0.273 (0.046)	0.273 (0.036)
Mean Platelet Volume (fL)	Baseline	Mean (SD)	10.23 (1.05)	10.32 (0.98)
	Week 48	Mean (SD)	11.89 (11.48)	10.28 (1.02)
		Low n (%)	8 (17.0%)	12 (25.5%)
		Normal n (%)	37 (78.7%)	35 (74.5%)
		High n (%)	2 (4.3%)	0

Parameters	Time	Statistics	HE (N = 47)	Placebo (N = 47)
**Biochemistry**				
Sodium (mmol/L)	Baseline	Mean (SD)	139.1 (2.2)	139.5 (2.2)
	Week 48	Mean (SD)	138.9 (2.1)	139.4 (2.0)
		Low, n (%)	2 (4.3%)	2 (4.3%)
		Normal, n (%)	45 (95.7%)	45 (95.7%)
Potassium (mmol/L)	Baseline	Mean (SD)	4.54 (0.60)	4.48 (0.36)
	Week 48	Mean (SD)	4.35 (0.25)	4.31 (0.24)
		Normal, n (%)	47 (100.0%)	47 (100.0%)
Chloride (mmol/L)	Baseline	Mean (SD)	103.1 (2.3)	104.0 (2.4)
	Week 48	Mean (SD)	103.0 (2.6)	103.7 (1.9)
		Low n (%)	2 (4.3%)	0
		Normal n (%)	45 (95.7%)	46 (97.9%)
		High n (%)	0	1 (2.1%)
Calcium (mmol/L)	Baseline	Mean (SD)	2.425 (0.099)	2.392 (0.087)
	Week 48	Mean (SD)	2.405 (0.108)	2.395 (0.095)
		Normal n (%)	45 (95.7%)	46 (97.9%)
		High n (%)	2 (4.3%)	1 (2.1%)
Inorganic Phosphorus (mmol/L)	Baseline	Mean (SD)	1.179 (0.115)	1.191 (0.115)
	Week 48	Mean (SD)	1.170 (0.123)	1.199 (0.103)
		Normal n (%)	46 (97.9%)	45 (95.7%)
		High n (%)	1 (2.1%)	2 (4.3%)
Magnesium (mmol/L)	Baseline	Mean (SD)	0.823 (0.051)	0.848 (0.049)
	Week 48	Mean (SD)	0.804 (0.055)	0.831 (0.056)
		Normal n (%)	47 (100.0%)	47 (100.0%)
Uric Acid (umol/L)	Baseline	Mean (SD)	256.6 (54.4)	266.7 (51.5)
	Week 48	Mean (SD)	270.9 (59.8)	283.7 (61.8)
		Normal n (%)	46 (97.9%)	44 (93.6%)
		High n (%)	1 (2.1%)	3 (6.4%)
Urea (mmol/L)	Baseline	Mean (SD)	4.72 (1.07)	5.46 (1.09)
	Week 48	Mean (SD)	4.84 (1.04)	5.48 (1.20)
		Normal n (%)	47 (100.0%)	45 (95.7%)
		High n (%)	0	2 (4.3%)
Creatinine (umol/L)	Baseline	Mean (SD)	66.7 (5.8)	68.7 (7.8)
	Week 48	Mean (SD)	68.6 (8.0)	71.0 (10.9)
		Normal n (%)	46 (97.9%)	44 (93.6%)
		High n (%)	1 (2.1%)	3 (6.4%)
Total Bilirubin (umol/L)	Baseline	Mean (SD)	10.33 (3.89)	10.06 (3.91)
	Week 48	Mean (SD)	9.79 (3.96)	10.19 (3.74)
		Normal n (%)	46 (97.9%)	46 (97.9%)
		High n (%)	1 (2.1%)	1 (2.1%)
Alkaline Phosphatase (IU/L)	Baseline	Mean (SD)	70.9 (16.6)	76.8 (18.7)
	Week 48	Mean (SD)	72.5 (15.7)	77.2 (18.8)
		Low n (%)	1 (2.1%)	0
		Normal n (%)	46 (97.9%)	47 (100.0%)
Aspartate Aminotransferase (IU/L)	Baseline	Mean (SD)	23.4 (4.9)	21.8 (6.7)
	Week 48	Mean (SD)	23.3 (4.9)	22.0 (8.9)
		Normal n (%)	47 (100.0%)	45 (95.7%)
		High n (%)	0	2 (4.3%)
Alanine Aminotransferase (IU/L)	Baseline	Mean (SD)	21.0 (9.3)	19.8 (8.0)
	Week 48	Mean (SD)	20.8 (7.3)	20.3 (12.1)
		Normal n (%)	47 (100.0%)	46 (97.9%)

Parameters	Time	Statistics	HE (N = 47)	Placebo (N = 47)
		High n (%)	0	1 (2.1%)
Gamma-Glutamyl Transferase (IU/L)	Baseline	Mean (SD)	24.0 (15.8)	23.4 (16.2)
	Week 48	Mean (SD)	24.0 (15.8)	20.9 (13.7)
		Low n (%)	0	2 (4.3%)
		Normal n (%)	39 (83.0%)	42 (89.4%)
		High n (%)	8 (17.0%)	3 (6.4%)
Total Proteins (g/L)	Baseline	Mean (SD)	71.0 (4.8)	69.5 (3.4)
	Week 48	Mean (SD)	71.2 (4.2)	69.9 (3.5)
		Low n (%)	0	1 (2.1%)
		Normal n (%)	46 (97.9%)	46 (97.9%)
		High n (%)	1 (2.1%)	0
Albumin (g/L)	Baseline	Mean (SD)	44.7 (2.3)	43.4 (2.0)
	Week 48	Mean (SD)	45.1 (2.2)	44.3 (1.7)
		Normal n (%)	47 (100.0%)	47 (100.0%)
Albumin/Globulin Ratio	Baseline	Mean (SD)	1.74 (0.24)	1.69 (0.18)
	Week 48	Mean (SD)	1.76 (0.25)	1.75 (0.18)
		Normal n (%)	43 (91.5%)	45 (95.7%)
		High n (%)	4 (8.5%)	2 (4.3%)
Globulin (g/L)	Baseline	Mean (SD)	26.2 (4.0)	26.1 (2.6)
	Week 48	Mean (SD)	26.1 (4.0)	25.6 (2.7)
		Low n (%)	1 (2.1%)	0
		Normal n (%)	45 (95.7%)	47 (100.0%)
		High n (%)	1 (2.1%)	0

Table S5. Plasma bone biomarkers at 48 weeks (change from baseline).

Parameter	Unit	Statistics	HE (n = 46)	Placebo (n = 47)	*p*
CTx	ng/ml	median (Q1; Q3)	0.037 (−0.009 ; 0.165) ***	0.071 (0.005 ; 0.164) ***	0.55
PINP	pg/ml	mean (SD)	360.6 (7680.0)	−1199.0 (7804.8)	0.25
Oc	pg/ml	mean (SD)	115.7 (7634.7)	183.1 (9067.6)	0.23
uOc	pg/ml	mean (SD)	−300.5 (1169.2)**	−281.8 (1376.4)	0.22
Sclerostin	pg/ml	mean (SD)	−78.0 (342.4)*	−110,8 (299.5)*	0.81
BALP	ng/ml	median (Q1; Q3)	−0.135 (−1.035; 0.318)	−0.001 (−0.454; 0.618)	0.16
TRAP5b	mIU/ml	median (Q1; Q3)	−0.105 (−0.229 ; 0.025) **	−0.079 (−0.281 ; 0.013) *	0.50
BALP / TRAP5b	ng/mIU	median (Q1; Q3)	0.304 (−1.816 ; 3.060)	1.146 (−0.759 ; 2.582) *	0.59

*$p < 0.05$, ** $p < 0.01$, *** $p < 0.001$ versus baseline

Table S6. Anthropometrics and blood efficacy parameters at 48 weeks (change from baseline).

Parameter	Unit	Statistics	n	HE	n	Placebo	*p*
Weight	kg	median (Q1; Q3)	47	0.50 (-0.70; 2.10)	47	0.40 (-1.50; 1.30)	0.137[2]
BMI	kg/m^2	median (Q1; Q3)	47	0.24 (-0.19; 0.82)	47	0.10 (-0.53; 0.51)	0.179[2]
Waist circumference	cm	median (Q1; Q3)	47	0.0 (0.0; 1.0)	47	0.0 (0.0; 1.0)	0.125[3]
Hip circumference	cm	median (Q1; Q3)	47	0.0 (0.0; 1.0)	47	0.0 (-1.0; 1.0)	0.143[3]
Waist-to-hip ratio	a.u.	mean (SD)	47	0.001 (0.012)	47	0.001 (0.013)	0.972[1]
Total Cholesterol	mmol/L	mean (SD)	46	0.08 (0.56)	45	0.16 (0.60)	0.510 [1]
LDL-Cholesterol	mmol/L	median (Q1; Q3)	46	−0.05 (−0.40; 0.30)	45	0.20 (−0.30 ; 0.40)	0.102 [3]
HDL-Cholesterol	mmol/L	median (Q1; Q3)	46	0.03 (−0.080; 0.150)	45	0.11 (0.020; 0.220)	0.170 [2]
Triglycerides	mmol/L	median (Q1; Q3)	46	0.045 (−0.090; 0.220)	45	0.01 (−0.240; 0.150)	0.770 [2]
Fasting Glucose	mmol/L	mean (SD)	45	0.04 (0.38)	44	0.04 (0.41)	0.956 [1]
Fasting Insulinaemia	mIU/L	median (Q1; Q3)	46	0.085 (−0.800; 1.300)	44	0.085 (−1.040; 1.160)	0.742 [2]
HbA1c	mmol/mol	mean (SD)	46	−0.9 (1.6)	44	−1.1 (2.2)	0.943 [1]
HOMA-IR	a.u.	median (Q1; Q3)	45	0.08 (−0.16 ; 0.31)	44	0.02 (−0.23 ; 0.33)	0.753 [2]
17-β Oestradiol	pmol/L	median (Q1; Q3)	47	0.0 (0.0 ; 0.0)	46	0.0 (0.0 ; 0.0)	0.872 [3]
Serum 25-OH D3	nmol/L	median (Q1; Q3)	47	20.0 (2.0; 29.0)	45	22.0 (6.0; 36.0)	0.254 [3]

[1] MMRM including analysis group, baseline value, visit and interaction group * visit with unstructured matrix of variance-covariance; [2] MMRM on logtransformed values including analysis group, baseline value, visit and interaction group * visit with unstructured matrix of variance-covariance; [3] Rank ANCOVA at each timepoint including analysis group and baseline value.

Table S7. Dietary intake and physical activity at 48 weeks (change from baseline).

Parameter		Unit	Statistics	HE (n = 47)	Placebo (n = 47)	*p*
**Dietary Intake**	Energy	kcal/d	median (Q1; Q3)	50.3 (−268.5; 430.6)	47.8 (−452.5; 297.0)	0.215 [1]
	Fat	g/d	median (Q1; Q3)	3.6 (−9.9; 26.2)	−7.3 (−21.8; 7.1)	0.048 [2]
	Carbohydrate	g/d	mean (SD)	17.2 (79.3)	2.5 (66.5)	0.246 [3]
	Protein	g/d	median (Q1; Q3)	−3.0 (−12.2; 19.8)	−2.6 (−20.0; 14.3)	0.299 [1]
	Fibre	g/d	mean (SD)	3.4 (10.9)	1.2 (8.8)	0.165 [3]
	Isoflavone	µg/d	median (Q1; Q3)	−7.0 (−34.8; 50.6)	1.2 (−25.4; 11.6)	0.101 [2]
	Calcium	mg/d	median (Q1; Q3)	5.9 (−224.1; 469.2)	−105.8 (−296.3; 131.9)	0.040 [2]
	Magnesium	mg/d	median (Q1; Q3)	11.0 (−52.1; 99.4)	7.1 (−76.9; 55.4)	0.422 [1]
	Phosphorus	mg/d	median (Q1; Q3)	37.9 (−295.9; 395.1)	−110.4 (−376.4; 230.6)	0.105 [1]
	Potassium	mg/d	median (Q1; Q3)	59.3 (−541; 882.6)	−3.8 (−577.9; 882.1)	0.659 [1]
	Sodium	mg/d	mean (SD)	174.9 (656.3)	−0.1 (640.3)	0.080 [3]
	Vitamin D	µg/d	median (Q1; Q3)	−0.2 (−1.5; 0.9)	−0.4 (−2.0; 0.2)	0.442 [2]
	Vitamin K1	µg/d	median (Q1; Q3)	20.6 (−19.8; 79.4)	11.8 (−17.3; 53.5)	0.343 [1]
	Vitamin K2	µg/d	median (Q1; Q3)	1.9 (−2.5; 4.6)	−0.4 (−4.5; 2.1)	0.015 [1]
**Physical activity**	Total PASE	a.u.	mean (SD)	−1.7 (56.1)	−19.5 (68.5)	0.563 [4]

[1] Rank ANCOVA at each timepoint including analysis group and baseline value; [2] ANCOVA on log-transformed values including analysis group and baseline value; [3] ANCOVA including analysis group and baseline value; [4] MMRM including analysis group, baseline value, visit and interaction group*visit with unstructured matrix of variance-covariance.

Table S8. SCFA concentrations at 24 weeks and 48 weeks (change from baseline).

		**HE**		**Placebo**	
SCFA, in μmol/g of dry weight fecal matter	statistics	Δ 24 wks	Δ 48 wks	Δ 24 wks	Δ 48 wks
acetate	median	4.3	9.9	19.3	23.8
	Q1	−22.4	−20.6	−0.8	5.5
	Q3	35.5	38.2	28.0	44.2
Propionate	median	1.2	2.5	2.2	5.0
	Q1	−8.8	−6.7	−3.2	0.5
	Q3	9.0	10.5	9.4	13.8
Butyrate	median	0.0	8.9	3.8	2.1
	Q1	−8.9	−13.6	−2.2	−7.7
	Q3	14.6	18.1	16.2	21.7
Valerate	median	0.0	2.3	3.1	2.4
	Q1	−5.5	−5.2	−1.5	−3.1
	Q3	7.1	8.5	15.8	9.4
Isobutyrate	median	0.6	0.0	0.3	0.9
	Q1	−1.4	−1.9	−0.8	−0.4
	Q3	2.8	2.8	3.7	4.5
Isovalerate	median	1.8	0.5	0.8	2.4
	Q1	−3.2	−2.4	−1.4	−1.1
	Q3	6.2	5.9	7.5	8.9
Caproate	median	1.1	1.2	4.5	1.7
	Q1	−1.2	−2.7	0.0	−1.7
	Q3	4.9	4.8	7.7	3.9
Isocaproate	median	0.0	0.0	0.0	0.0
	Q1	0.0	0.0	0.0	0.0
	Q3	1.0	0.3	1.2	0.0
Total SCFA	median	11.23	25.4	27.2	36.5
	Q1	−46.4	−38.6	0.0	0.0
	Q3	68.4	71.0	78.2	92.0
